# Biofortification of Three Cultivated Mushroom Species with Three Iron Salts—Potential for a New Iron-Rich Superfood

**DOI:** 10.3390/molecules27072328

**Published:** 2022-04-04

**Authors:** Sylwia Budzyńska, Marek Siwulski, Monika Gąsecka, Zuzanna Magdziak, Pavel Kalač, Przemysław Niedzielski, Mirosław Mleczek

**Affiliations:** 1 Department of Chemistry, Poznan University of Life Sciences, Wojska Polskiego 75, 60-625 Poznań, Poland; monika.gasecka@up.poznan.pl (M.G.); zuzanna.magdziak@up.poznan.pl (Z.M.); miroslaw.mleczek@up.poznan.pl (M.M.); 2 Department of Vegetable Crops, Poznan University of Life Sciences, Dąbrowskiego 159, 60-594 Poznań, Poland; marek.siwulski@up.poznan.pl; 3 Department of Applied Chemistry, Faculty of Agriculture, University of South Bohemia, 370 04 České Budějovice, Czech Republic; kalac@zf.jcu.cz; 4 Faculty of Chemistry, Adam Mickiewicz University in Poznań, Uniwersytetu Poznańskiego 8, 61-614 Poznań, Poland; pnied@amu.edu.pl

**Keywords:** functional food, deficiency, malnutrition, organic acids, phenolic acids, *Pholiota nameko*, *Pleurotus eryngii*, *Pleurotus ostreatus*, supplementation

## Abstract

Mushrooms fortified with iron (Fe) can offer a promising alternative to counter the worldwide deficiency problem. However, the factors that may influence the efficiency of fortification have not yet been fully investigated. The aim of this study was to compare the effects of three Fe forms (FeCl_3_ 6H_2_O, FeSO_4_ 7H_2_O, or FeHBED) in three concentrations (5, 10, or 50 mM) for three mushroom species (*Pleurotus eryngii*, *P. ostreatus,* or *Pholiota nameko*) on their chemical composition, phenolic compounds, and organic acid production. The most effective metal accumulation of all the investigated species was for the 50 mM addition. FeCl_3_ 6H_2_O was the most favorable additive for *P. eryngii* and *P. nameko* (up to 145 and 185% Fe more than in the control, respectively) and FeHBED for *P. ostreatus* (up to 108% Fe more than in control). Additionally, *P. nameko* showed the highest Fe accumulation among studied species (89.2 ± 7.51 mg kg^−1^ DW). The creation of phenolic acids was generally inhibited by Fe salt supplementation. However, an increasing effect on phenolic acid concentration was observed for *P. ostreatus* cultivated at 5 mM FeCl_3_ 6H_2_O and for *P. eryngii* cultivated at 5 mM FeCl_3_ 6H_2_O and 5 mM FeSO_4_ 7H_2_O. In the case of organic acids, a similar situation was observed. For *P. ostreatus*, FeSO_4_ 7H_2_O and FeHBED salts increased the formation of the determined organic acids in fruiting bodies. *P. eryngii* and *P. nameko* were characterized by a much lower content of organic acids in the systems supplemented with Fe. Based on the obtained results, we recommend starting fortification by preliminarily indicating which form of the element is preferred for the species of interest for supplementation. It also seems that using an additive concentration of 50 mM or higher is most effective.

## 1. Introduction

Iron (Fe) is a transition metal and a trace element that is indispensable for all human cell life in a myriad of physiological processes in the body [1,2,3]. It is an essential component of numerous proteins (hemoglobin, myoglobin, cytochromes, and enzymes) participating in vital metabolic functions such as oxygen transport, oxidative energy production, mitochondrial respiration, harmful reactive oxygen species (ROS) inactivation, and deoxyribonucleic acid (DNA) synthesis and repair [4]. An impressive illustration of this crucial role of Fe was made by Muckenthaler et al. [5], according to which more than two quadrillion Fe atoms are required every second to produce 200 billion red blood cells every day by a human to maintain adequate erythropoiesis. Unfortunately, deficiency of Fe (iron deficiency, ID) is one of the predominant nutritional disorders that lead to cellular metabolism perturbations. It is the most significant contributor to anemia (iron deficiency anemia, IDA) as well as other physical and neurodevelopmental morbidities [6,7]. It impairs exercise capacity, worsens the quality of life, increases hospitalization rate and mortality [1,8]. Confirming the above, the World Health Organization (WHO) has recognized IDA as the most common nutritional deficiency worldwide, with 30% of the population being affected. It is estimated that around 800 million children and women worldwide are anemic, mainly owing to ID (48% of children between 5 and 14 years and 52% of pregnant women in developing countries) [9,10].

Various strategies to enhance the Fe content and bioavailability in food crops to minimize metal deficiency have been investigated [11,12]. Food Fe biofortification is probably the most cost-effective, practical, and sustainable long-term solution [13]. It is necessary to select the food product to be fortified because of differences in Fe accumulation efficiency. It is also crucial to consider bioavailability when selecting the Fe fortification compound because the success of the enrichment depends heavily on its absorbability [14]. Iron is regarded as the most challenging micronutrient to add to fortified food due to its various chemical forms’ different properties [3]. Former and recent studies have demonstrated that selected plants can be successfully enriched with Fe, e.g., beans [15], cowpea [16], pearl millet [17], rice [18], soybean [19], and wheat [20].

As more and more mushroom species are cultivated for commercial purposes and as they have excellent bioaccumulation capabilities, the mushroom can be a promising alternative for enriching food with minerals essential to human health [21,22,23]. The search for species capable of Fe supplementation is becoming increasingly popular. For example, Vieira et al. [24] described *Pleurotus ostreatus* enriched with ferrous sulfate (FeSO_4_, 0.8 mg kg^−1^). Unfortunately, substrate enrichment with the mineral promoted a lower content of Fe in cultivated mushrooms than in control (111.53 and 149.14 mg kg^−1^ dry weight (DW), respectively). Additionally, the authors reported a reduction in the antioxidant activity of *P. ostreatus* enriched with Fe. Inhibitory interaction between the fungi and Fe was also shown by Ogidi et al. [25]. They studied *P. ostreatus*, *P. cornucopiae*, *P. diamor*, *P. pulmonarius,* and *P. djamor v. roseus*. The mycelia growth rate and mushroom biomass production decreased as Fe concentration increased in the substrate during the study. The estimated Fe content in the cultivated *Pleurotus* species was 37.8–96.6 mg kg^−1^ DW. The influence of different variables (pH: 4.5 or 6.5; FeSO_4_ concentration: 50 or 150 mg L^−1^; and carbon and nitrogen sources) on *P. ostreatus* growth and Fe bioaccumulation was also studied by Almeida et al. [26]. The authors concluded that the most critical variable of bioaccumulation was Fe concentration in the substrate. They showed that a concentration equal to or higher than 175 mg Fe L^−1^ in the cultivation medium inhibited mycelial growth. In the described study, Fe bioaccumulation in the mycelium was 507–3616 mg kg^−1^. Despite the difficulties in supplementation, all the authors stated that Fe-enriched mushrooms are a potential alternative to producing non-animal sources of Fe.

Different organic compounds (glucans, phenolic compounds, organic acids, etc.) present in mushrooms may benefit human health [27,28,29]. Phenolic compounds, including phenolic acids, are important secondary metabolites of mushrooms, demonstrating antioxidative and anti-inflammatory properties [30,31,32,33]. The antioxidative actions of phenolic acids against cell damage can be accomplished in different ways such as donation of electrons, thereby neutralizing reactive oxygen species (ROS), chelation elements which can generate ROS (e.g., Fe and copper (Cu)), or inhibition in the formation of free radicals through the inhibition of oxidases including lipoxygenase, cyclooxygenase, and NADH oxygenase [34]. The bioactive properties of organic acids, namely citric, ketoglutaric, malic, tartaric, succinic, and fumaric acids, including their abilities to delocalize the electric charge that comes from free radicals, may have a protective role against various diseases due to their antioxidant activity [35,36] and development of immune-stimulating activity [37]. In addition, organic acids regulate the availability of metal ions (both in terms of their environmental migration and ecological toxicology) through chelation, complexation, and adsorption reactions [38]. The composition and content of phenolic acids as well as organic acids are diverse and depend on the species [31,39,40,41]. Quantitative analyses of organic and phenolic acids are essential for understanding the correlation between chemical composition and biological activity.

The aim of this study was to show the potential of three cultivated mushroom species (*P. eryngii*, *P. ostreatus*, and *Pholiota nameko*) growing on substrates enriched with three different Fe salts in three different concentrations for the accumulation of Fe and selected other metals, biomass crop, and the creation of phenolic compounds and organic acids. As an additional goal, we attempted to propose the most effective concentration of Fe additive for fortification.

## 2. Results

### 2.1. Morphology and Biomass of Fruit Bodies

**Figure 1:**
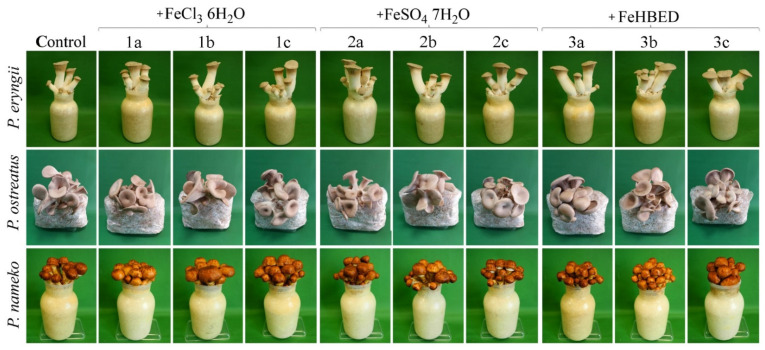
Macroscopic characteristics of control and treated fruit bodies growing on substrate enriched with FeCl_3_ 6H_2_O (1), FeSO_4_ 7 H_2_O (2), and FeHBED (3) in concentrations of 5 (a), 10 (b), and 50 (c) mM.

**Figure 2:**
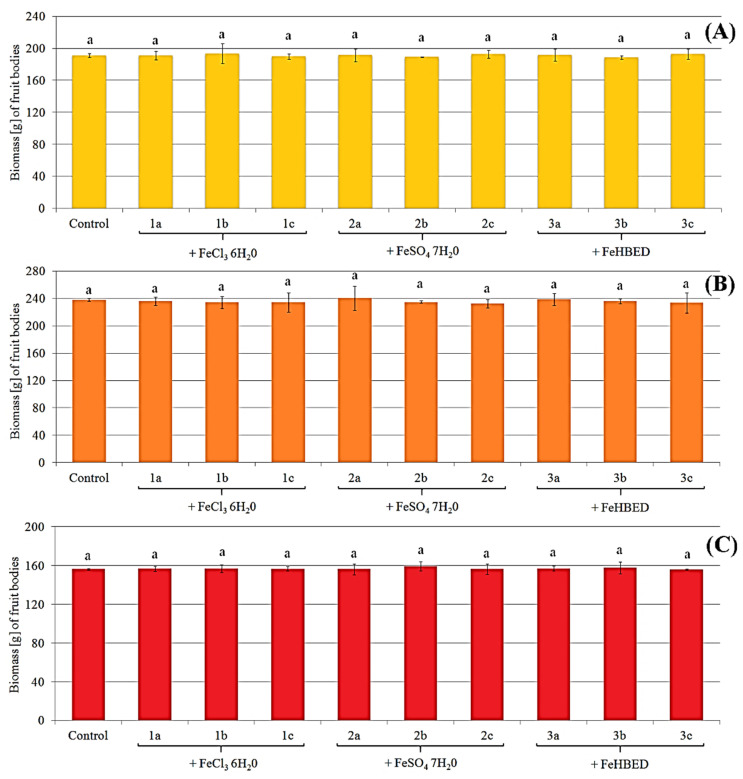
Yield (g) of *P. eryngii* (**A**), *P. ostreatus* (**B**), and *P. nameko* (**C**) exposed to particular experimental systems. *n* = 3, identical lower cases (^a^, ^b^…) denote non-significant differences between mean mushroom yield growing in particular experimental systems according to post-hoc Tukey’s HSD test.

There were no differences in size and color between control fruit bodies and treated specimens growing on substrate enriched with different concentrations of particular Fe salts (Figure 1). The same tendency was also observed for biomass. Increased concentration of Fe did not cause a significantly higher or lower crop of mushrooms (Figure 2).

The mean biomass of control *P. eryngii* was 191 ± 2 g with a range of 5 g. In the case of treated fruit bodies growing under systems 1a–c, 2a–c, and 3a–c, the mean biomass was: 192 ± 7, 191 ± 5, and 191 ± 6 g, respectively (Figure 2A). The ranges for these groups were 24, 15, and 17 g, respectively, which confirms the similar crops of *P. eryngii* independently of the experimental system. High similarity in the crop of the control and the treated fruit bodies was also observed for the rest of the mushroom species. The mean biomass of control *P. ostreatus* was 151 ± 1 g. However, the mean value for fruit bodies under 1a–c, 2a–c, and 3a–c systems was the same (157 g) with the ranges: 7, 13, and 12 g, respectively (Figure 2B). The highest biomass values were characterized by fruit bodies of *P. nameko* being 238 ± 2 g for the control, while for the treated systems 1a–c, 2a–c, and 3a–c, mean values of biomass were: 235 ± 9, 236 ± 10, and 236 ± 9 g with ranges: 27, 33, and 29 g, respectively (Figure 2C).

### 2.2. Content of Iron in Fruit Bodies

**Figure 3:**
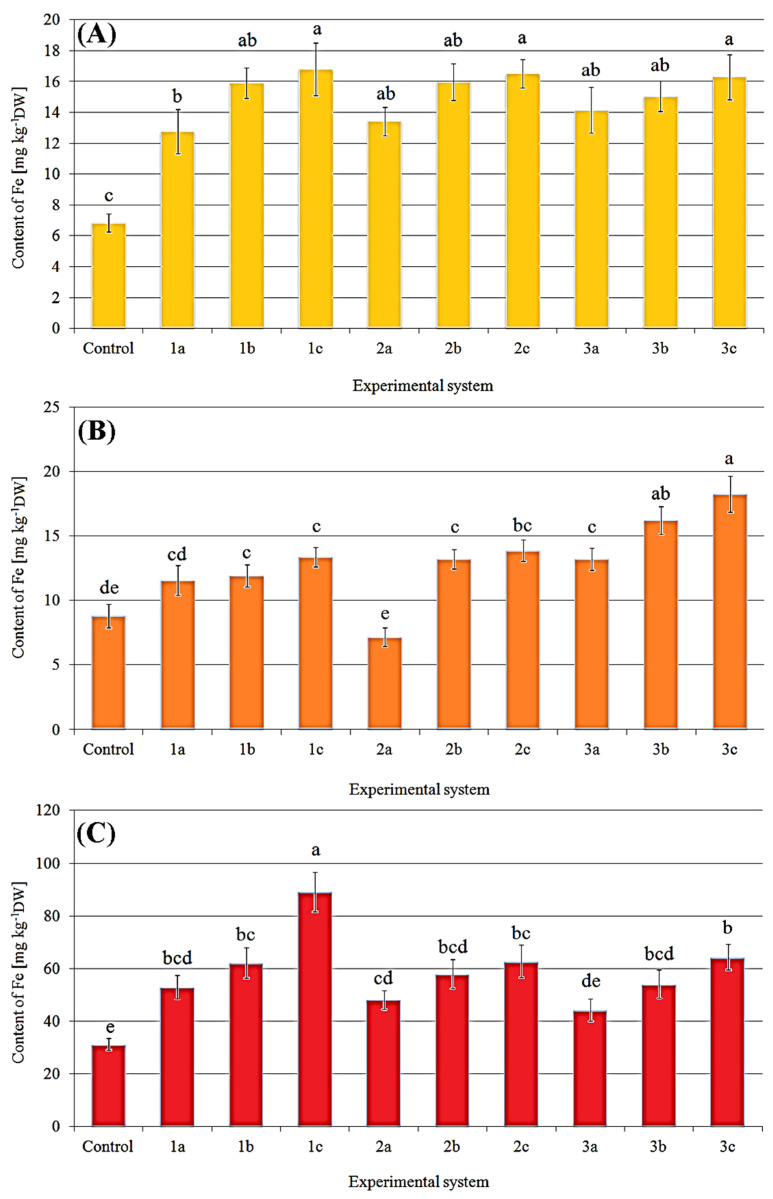
Content of Fe (mg kg^−1^ DW) in *P. eryngii* (**A**), *P. ostreatus* (**B**), and *P. nameko* (**C**) in the first crop exposed to particular experimental systems. *n* = 3, identical lower cases (^a^, ^b^…) denote non-significant differences between mean content of Fe in fruit bodies growing in particular experimental systems according to post-hoc Tukey’s HSD test.

The content of Fe in treated bodies of *P. eryngii* was significantly higher than the control (6.85 ± 0.59 mg kg^−1^ DW) each time (Figure 3). Generally, the increase of Fe content in substrate caused the increased accumulation in fruit bodies. The highest contents of Fe in fruit bodies growing under the 1c, 2c, and 3c systems (16.8 ± 1.71; 16.5 ± 0.93; and 16.3 ± 1.46 mg kg^−1^ DW) were observed, corresponding to 245, 241, and 238% of the control, respectively (Figure 3A). The content of Fe in *P. ostreatus* fruit bodies treated with Fe salts was higher than in the control except for mushrooms growing under the 2a systems (Figure 3B). The highest content of Fe (18.3 ± 1.41 mg kg^−1^ DW being 208% of control) was recorded for *P. ostreatus* treated with the highest concentration of FeHBED (3c system). An increased concentration of FeCl_3_ (1a–c systems) in the substrate did not lead to a higher content of this metal in fruit bodies, while for the 2a–c and 3a–c systems, an apparent increase of Fe content was observed. Fruit bodies of *P. nameko* were the most enriched among the three studied mushroom species (Figure 3C). The most effective accumulation of Fe was noted in *P. nameko* growing under the 1c system (89.2 ± 7.51 mg kg^−1^ DW). The content of Fe in these fruit bodies was almost 285% of the control (31.3 ± 2.18 mg kg^−1^ DW). An increase in Fe content with this metal present in the substrate was also observed for this mushroom species.

**Figure 4:**
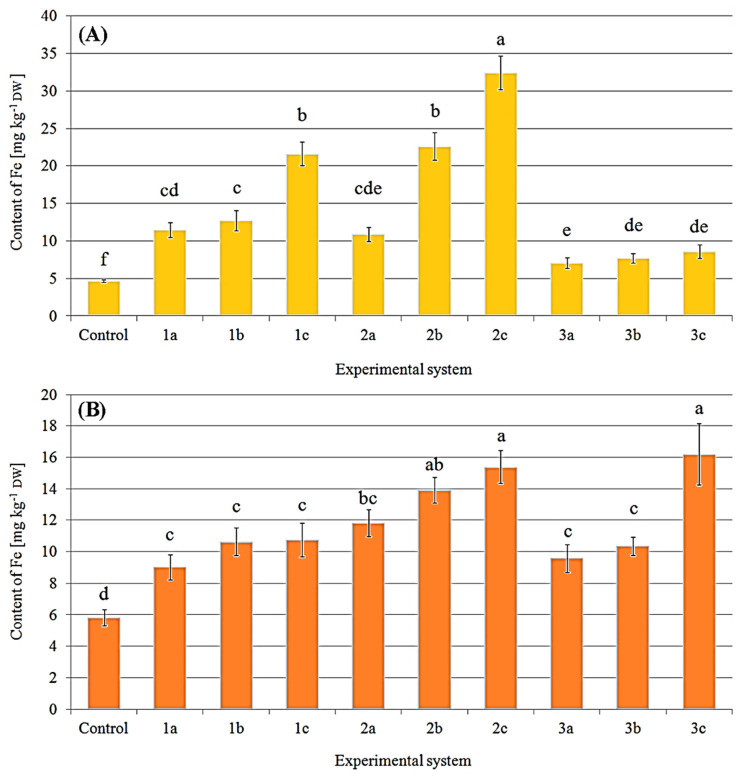
Content of Fe (mg kg^−1^ DW) in *P. eryngii* (**A**) and *P. ostreatus* (**B**) in the second crop exposed to particular experimental systems. *n* = 3, identical lower cases (^a^, ^b^…) denote non-significant differences between mean content of Fe in fruit bodies growing in particular experimental systems according to post-hoc Tukey’s HSD test.

It is worth underlining that the above results considered the first crop. In the case of *P. eryngii* and *P. ostreatus,* the second crop was also collected. Significantly higher content of Fe in all treated *P. eryngii* and *P. ostreatus* was observed compared to the control (4.64 ± 0.18 mg kg^−1^ DW) (Figure 4). The highest content of Fe was found (32.5 ± 2.26 mg kg^−1^ DW) in *P. eryngii* growing under the 2c system, which corresponds to 702% of the control (Figure 4A). The apparent increase in Fe content in *P. ostreatus* fruit bodies with the increase in the concentration of this metal in the substrate was recorded for all experimental systems except 2a–c, where the content of Fe was almost the same (Figure 4B).

### 2.3. Content of Selected Elements in Mushrooms

Supplementation of mushroom species with Fe salts influenced the content of this and other selected metals (Ca, K, Mg, Mn, Na, and S) (Table 1, Table 2 and Table 3). Generally, an increase in the element mentioned above depends on the Fe concentration in the applied salts. For most elements, a significantly higher content was observed in mushrooms growing on substrates enriched with 10 or 50 mM of Fe than in the control. The content of Ca in *P. eryngii* (I crop) was significantly higher than in the control for all treated systems while for the second crop, it was only the case for 1a–c, 2c, and 3b–c systems (Table 1). In the case of *P. ostreatus* (I crop), a significantly higher Ca content in mushrooms under 2a–c and 3a–c than in control was recorded, while for the second crop, this tendency was observed for all treated systems. *Pholiota nameko* was characterized by a significantly higher Ca content in all systems enriched with FeCl_3_ (1a–c), 2b–c, and 3c systems (Table 3).

The content of K was significantly higher in *P. eryngii* growing under the 1b–c, 2b–c, and 3a–c systems than in the control (I crop). In comparison, for the second crop of this mushroom species, a higher content was recorded under the 1b–c and 2c systems only (Table 1). In the case of *P. ostreatus* from the I crop, a significantly higher K content was observed for mushrooms under the 2c and 3a–c systems, while for the second crop, it was observed for fruit bodies growing under the 2a–c and 3b–c systems. In *P. nameko,* there were no significant changes in K content with the addition of Fe salts(Table 3). The content of Mg in the treated fruit bodies of *P. eryngii* and *P. nameko* was significantly higher than in the control each time (I and II crop) when mushrooms were grown on substrate enriched with salts in concentrations of 10 or 50 mM (Table 1 and Table 3). This tendency was not observed for *P. ostreatus* whereas for the I crop, a significantly higher content of Mg was observed for mushrooms under 1b–c and 3a–c compared to the control. It is worth underlining that for the second crop, a significantly higher content of Mg was only recorded for fruit bodies under the 2a–c and 3c systems.

All fruit bodies of *P. eryngii* treated with particular Fe salts (I crop) were characterized by a significantly higher content of Mn. In contrast, a higher content of this metal was only noted in mushrooms treated with FeCl_3_ (1a–c systems) for the second crop. The same observation was accurate for *P. nameko*, where a significantly higher content of Mg was observed for mushrooms treated with 1a–c compared to the control. Exposure of *P. ostreatus* to FeCl_3_ (1a–c systems) or FeSO_4_ (2a–c systems) caused no significant changes in Mn content in mushrooms. In contrast, the addition of FeHBED (3a–c systems) led to a significantly higher metal content in collected fruit bodies (Table 2). The content of Na in treated *P. eryngii* fruit bodies (I crop) under the 2b–c and 3a–c systems was significantly higher than in the control, while for the second crop, it was only the case for bodies collected from the 1a–c and 2c experimental systems. Significantly higher Na content was observed both for *P. ostreatus* from the I and II crops growing under the 1b–c, 2b–c, and 3a–c systems, as was the case for *P. nameko* growing on substrates enriched with FeCl_3_ (1a–c systems) and FeHBED. The content of S was the same in both treated and control mushrooms of *P. eryngii* (I crop) and *P. nameko*. In contrast, for the rest of the mushroom species and crops, a significantly higher S content was recorded for mushrooms growing on substrate enriched with FeSO_4_ (2a–c systems) and FeHBED (3a–c systems) than in the control.

Of the Fe salts used in the experiment, FeHBED caused the most remarkable changes in the content of studied elements. The highest mean content of Ca was found in *P. nameko* fruit bodies, Na, and S (3060, 339, and 6230 mg kg^−1^ DW, respectively) and the lowest mean content of K (11,300 mg kg^−1^ DW) in all treated systems. Generally, for most of the studied elements, the content in mushrooms from I crop was higher than from II crop.

### 2.4. Content of Phenolic Compounds in Supplemented Mushrooms

Supplementation significantly affected the phenolic acid profile (Table 4). Two-way ANOVA confirmed the effect of both salt and its concentration in all analyzed mushrooms. Nine phenolic acids were quantified in the control of *P. ostreatus*: gallic, 2,5-dihydroxybenzoic (2,5-DHBA), 4-hydroxybenzoic (4-HBA), vanillic, caffeic, syringic, chlorogenic, ferulic, and sinapic, with only 2,5-dihydroxybenzoic and sinapic acid achieving the highest level.

The content of single phenolic acids ranged from 0.470 to 75.3 µg g^−1^ DW for ferulic and 4-HBA, respectively. Supplementation affected the profile and the concentration of the acids compared to the control. The highest content of phenolic acids (rising to 108 µg g^−1^ DW for 4-HBA) and the sum of all detected acids (an increase from 194 to 295 µg g^−1^ DW) was confirmed for fruiting bodies supplemented with FeCl_3_ at 5 mM. However, 2,5-DHBA was not detected in all supplemented fruiting bodies. The addition of a higher concentration of FeCl_3_ leads to a reduction in the content of acids, including a lack of some acids in the profile (chlorogenic and sinapic acids). The addition of FeSO_4_ and FeHBED reduced the content of the acids compared to the control and supplementation with FeCl_3_ at 5 mM. However, the profile of supplemented *P. ostreatus* became rich in *p*-coumaric acid in nearly all supplemented combinations.

The content of phenolic acids in fruit bodies of *P. eryngii* ranged from 0.570 to 6.99 µg g^−1^ DW, and the profiles contained gallic, vanillic, caffeic, syringic, *p*-coumaric, chlorogenic, ferulic, and sinapic acids in the control. Only chlorogenic, ferulic, and sinapic acids contents were higher in the control compared to the supplemented mushrooms. Supplementation of FeCl_3_ reduced the content of nearly all detected acids apart from gallic acid at 5 mM and *p*-coumaric at 10 mM. None of the determined phenolic acids was detected at 50 mM of FeCl_3_. The addition of 5 mM FeSO_4_ elevated the content of the vanillic, syringic, and chlorogic sum of acids. The addition of 10 and 50 mM of FeSO_4_ resulted in a reduction in the content of phenolic acids. The supplementation of FeHBED also led to a reduction in the acid content. At 10 and 50 mM of FeHBED, most analyzed acids were not detected. The sum of phenolic acids increased under 5 mM FeCl_3_ and 5mM FeSO_4_ from 21.9 to 26.6 and 36.9 µg g^−1^ DW, respectively.

The profile of phenolic acids in *P. nameko* was very poor. Only gallic and trans-cinnamic acids were found in the control reaching the highest values (6.69 and 12.8 µg g^−1^ DW, respectively). The addition of FeCl_3_ reduced the content of the acids, although 4-HBA was additionally detected. Gallic acid was not found after the addition of FeSO_4_ and FeHBED. The addition of all salts significantly decreased the sum of quantified acids from 19.5 µg g^−1^ DW in the control even up to 0.243 µg g^−1^ DW for 10 mM FeSO_4_.

### 2.5. Content of Organic Acids in Supplemented Mushrooms

The organic acid profile and content of enriched mushrooms were characterized by significant variation between particular *Pleurotus* mushroom species. Supplementation using Fe salts significantly affected the creation of organic acids. Two-way ANOVA confirmed the effect of both salt and the concentration of the organic acids in all analyzed mushroom samples.

Supplementation with Fe salts significantly influenced the profile and content of the analyzed organic acids determined in *P. ostreatus* fruiting bodies. Quinic acid was the main acid in the control system, but it was below the detection limit (bDL) in all samples supplemented with Fe salts. The content of acetic acid, the second main acid in the control samples, was at a comparable content and/or significantly reduced compared to the control fruit bodies. The content of malic acid in the analyzed samples of *P. ostreatus* was characterized by significant variability, resulting from both the added salt concentration and the form of Fe salt used. In the case of the FeCl_3_ salt, more than a two-fold increase in the content was determined compared to the control (0.630 in the control system and 1.44 µg g^−1^ for the 5 mM FeCl_3_ concentration), while for the remaining systems with a higher concentration of this salt, the amount of malic acid was below the detection level. In fruiting bodies supplemented with FeSO_4_ salt at a concentration of 5 mM, malic acid was below the detection level, and for the 5 and 10 mM system, its content was 0.870 and 19.6 µg g^−1^, respectively. The highest content of malic acid in *P. ostreatus* fruiting bodies was determined for FeHBED salt, where for a concentration of 10 and 50 mM of this salt, its content was 8.11 and 17.6 µg g^−1^ of malic acid. The remaining acids were detected sporadically or to a much lower content without significant changes. However, it should be emphasized that the highest sum of all acids detected in *P. ostreatus* was confirmed for fruiting bodies supplemented with FeHBED salt.

The fruiting bodies of *P. eryngii* were generally characterized by a significantly lower content of all analyzed organic acids compared to *P. ostreatus* species. *P. eryngii* was characterized by a similar acid content in the control system and the systems supplemented with FeCl_3_, FeSO_4_, and FeHBED at a concentration of 5 mM (35.3, 33.4, 35.7, and 39.4 µg g^−1^, respectively), while in the systems where the Fe content was 10 and 50 mM, a significant decrease in their content was determined compared to the control system (even to the bDL level for the FeCl_3_ salt). The presence of such acids as oxalic, malic, malonic, citric, and acetic was determined in *P. eryngii* fruiting bodies. As in the case of *P. ostreatus*, the malic acid content showed high variability, with the highest content obtained in the control system (10.7 µg g^−1^). Supplementation with FeCl_3_ and FeHBED salts resulted in a significant decrease of malic acid in each concentration of added salts except for the system with the addition of FeSO_4_ salts, where malic acid at a concentration of 5 mM was below the detection limit, although at 10 and 50 mM their content increased (5.32 and 2.28 µg g^−1^, respectively)—but this was still lower than in the control system. The content of acids in the fruiting bodies of *P. eryngii* for all tested Fe salts added at a concentration of 5 mM did not cause any major changes in the profile and content of the analyzed acids compared to the control. It is worth noting that in the systems where the Fe salt concentration was 10 and 50 mM, the content was characterized by a significant decrease (Table 5).

The profile and content of organic acids in *P. nameko* fruit body samples were very poor. The highest contents and composition of analyzed acids were obtained in the control where oxalic, malonic, citric, acetic, and fumaric acids were detected (16.3 µg g^−1^ DW). Supplementation using all Fe salts at 5, 10, and 50 mM resulted in a significantly lower content of all detected acids, simultaneously with bDL, in the case of samples supplemented by 5 and 50 mM FeSO_4_ and 10 mM FeHBED (Table 5).

## 3. Discussion

Iron deficiency is commonly a result of inadequate bioavailable Fe dietary intake and affects people of all ages [42]. The importance of improving Fe nutrition is receiving increased attention. Innovative therapies can efficiently recuperate normal serum transferrin saturation and concentration of indispensable hemoglobin but may cause various side effects [43,44]. The delivery of bioavailable nutrients from functional foods (called superfoods) fortified with Fe is a promising solution [45,46].

Mushrooms can bioaccumulate several metals of nutritional and pharmacological importance. With the addition of antioxidant and anti-inflammatory properties, it seems that they are promising candidates for functional food [47,48,49,50,51,52]. Reports of attempts to supplement mushrooms with Fe can be found increasingly in the literature. Meniqueti et al. [53] described Fe accumulation in the mycelial biomass of *Lentinus crinitus* cultivated in liquid malt extract with Fe in addition to obtaining 10, 20, 30, 40, 50, 60, 70, 80, 90, or 100 mg L^−1^ concentrations. Furthermore, the authors checked the efficiency of Fe accumulation during the growth of this species on agro-industrial byproducts (sugar cane and soybean molasses). The research mentioned above also evaluated Fe accumulation capacity in the vegetative mycelium of five edible and medicinal mushroom species [54]. *Agaricus subrufescens* Peck, *Ganoderma lucidum* (Curtis) P. Karst., *Pleurotus eryngii* (DC.) Quél., *P. ostreatus* (Jacq.) *P. Kumm.*, and *Schizophyllum commune* Fr. grown in malt extract with 10, 20, 30, 40, 50, 60, 70, 80, 90, and 100 mg L^−1^ Fe concentration. Oyetao et al. [55] examined *P. pulmonarius* supplementation with Fe. Biological efficiency, nutrient contents, and antioxidant activity in this mushroom species when 500 mg kg^−1^ Fe was added to a substrate-containing rice bran and maize stalk were described. Iron biofortification and availability from *L. crinitus*, *G. lucidum*, *Schizophyllum commune*, *P. ostreatus*, *P. eryngii*, *L. edodes,* and *A. subrufescens* grown in two different culture media (malt extract with 0.116 mg L^−1^ or sugarcane molasses with 91.2 mg L^−1^) were described by Scheid et al. [56]. A comparison of Fe bioaccumulation in mycelial biomass of *Letinula edodes*, *P. eryngii*, *P. ostreatus,* and *S. commune* grown in malt extract agar with 50 mg L^−1^ enrichment was made by Umeo et al. [57]. Evaluation of Fe content in *P. ostreatus* grown on the sugar cane (*Saccharum offiinarum* L.) bagasse with the addition of 0.5, 1, 2, 5, or 10 mg kg^−1^ Fe was performed by Condé et al. [58]. Mycelial growth, biomass production, and Fe uptake by *Pleurotus* species (*P. cornucopiae*, *P. djamor*, *P. ostreatus*, and *P. pulmonarius*) grown on a substrate based on *Urochloa decubens* with the addition of 500 or 1000 mg kg^−1^ Fe were described by Ogidi et al. [25]. Vieira et al. [24] investigated antioxidant activity as well as the phenolic and metal content of *P. ostreatus* grown on coffee husk enriched with Fe (0.800 mg kg^−1^).

Based on the available literature, it can be unequivocally concluded that the most critical variable for effective accumulation in mushrooms is Fe concentration in the substrate. In all of the manuscripts cited above, the researchers decided to add the metal in the form of iron (II) sulphate (VI) (FeSO_4_) or its hydrated form (FeSO_4_ 7H_2_O) in different content and concentrations. These results significantly expand our knowledge of mushroom supplementation with this metal. However, to further develop this issue, it is necessary to determine how we can maximize the increase in bioaccumulation potential. By obtaining as much value as possible, we are able to obtain iron-rich mushrooms that can be used as superfoods. Due to the fact that Fe exists in various chemical forms that may exhibit completely different properties during supplementation, the results presented in our study included a comparison of the effects of different forms of Fe (FeCl_3_ 6H_2_O, FeSO_4_ 7H_2_O, and FeHBED) in different concentrations (5, 10, or 50 mM, which correspond to 280, 560, or 2800 mg L^−1^) on different species of fungi. To the best of our knowledge, this is the first study to investigate the effect of a factor other than the concentration of one form of metal in the substrate.

In our experiment, we did not find any significant changes in the morphology and biomass production of the studied mushrooms from all systems. The obtained results clearly showed that the used concentrations of particular Fe salts neither stimulated nor inhibited fruit body crops. The same observation was made by the above mentioned Oyetayo et al. [55], where a 500 mg kg^−1^ addition did not reduce *P. pulmonarius* yield. No significant mushroom growth inhibition at lower concentrations was also described by Meniqueti et al. [53] (up to 70 mg L^−1^) and Almeida et al. [26] (up to 150 mg L^−1^). An opposite relationship was noted by Vieira et al. [24], where a decrease in Fe content in the fruit body was recorded when the substrate was enriched with 0.180 mg kg^−1^ FeSO_4_. Additionally, Meniqueti et al. [54] found that the metal content of the mushroom species they studied responded differently to Fe content added to the culture medium.

In general, for the vast majority of systems, adding each of the individual Fe forms to the substrate resulted in an increased content of this metal in the mushrooms compared to the control. For systems with the addition of FeSO_4_ 7H_2_O, the form used by other researchers, the highest accumulation increased by +185, +145, and +52% compared to the control (for *P. nameko*, *P. eryngii* and *P. ostreatus*, respectively). This is similar to the observations from all the papers cited above. However, in more detail, when we focused on the changes in accumulation as a function of the added concentration of this metal form, we found that the influence was no longer so predictable. In our experiment, the highest increases mentioned above were determined for the highest, 2800 mg L^−1^ FeSO_4_ 7H_2_O, addition for all species. In the study of Meniqueti et al. [53], the highest, a 9000-fold increase of Fe mycelial accumulation compared to the control, was observed for 90.0 mg L^−1^ FeSO_4_ 7H_2_O addition. However, lower (10–80 mg L^−1^) or higher (100 mg L^−1^) quantities of metal were also added to the substrate. According to Meniqueti et al. [54], the greatest Fe contents (from 4000 to 13,000-fold higher) in the mycelial biomass were obtained for the highest used metal concentration (100 mg L^−1^ FeSO_4_ 7H_2_O). In Condé et al. [58], the highest average Fe content was determined when the middle addition (1 mg kg^−1^ FeSO_4_) was supplemented. Almeida et al. [26] found that when 50 to 300 mg L^−1^ FeSO_4_ was added to the medium, the highest accumulation was obtained for 250 mg L^−1^.

The problem becomes even more complicated when we consider the various forms of metal that can be added to the substrate. In our experiment, the addition of inorganic iron (III) salt, inorganic iron (II) salt, and organic chelated iron (I) salt was compared. A common observation for all systems was the highest accumulation of Fe in fruit bodies of all species when the highest concentration of individual forms, 50 mM (2800 mg L^−1^), was added to the substrate. In the case of *P. eryngii*, the highest bioaccumulation was achieved when FeCl_3_ was added to the substrate, lower under FeSO_4_ 7H_2_O, and the lowest for FeHBED. For *P. ostreatus*, the highest content was found for the FeHBED system, followed by the FeSO_4_ 7H_2_O, and the lowest for FeCl_3_ addition. On the other hand, for *P. nameko*, the highest accumulation was determined for FeCl_3_ supplementation, lower for FeHBED, and the lowest for FeSO_4_ 7H_2_O. Therefore, it can be stated that Fe bioaccumulation efficiency is significantly influenced not only by metal concentration in the substrate and the mushroom species but also by the form of the metal used in the additive, which we aimed to demonstrate in this study.

Due to the lack of literature on mushroom supplementation with different Fe forms, it is difficult to compare the results with other studies. However, in research on the supplementation of other food products, the indisputable effect of the form of Fe present in the medium on which the supplemented plants grew was confirmed. In their review, Connorton and Balk [59] described Fe biofortification of staple crops in detail. The authors paid particular attention to the increase in the efficiency of this process due to the chelation of Fe forms present in the medium. The importance of Fe mobilization in plants through chelating with citrate, phytosiderophores, nicotiamine, mugineic acid, and in the form of free Fe ions was summarized by Shumayla et al. [60]. An indication of Fe-EDDHA as a more effective additive than FeSO_4_ at various concentrations (25, 50, and 100 µM) for Fe biofortification, antioxidant response, yield, and nutritional quality in green beans was demonstrated by Sida-Arreola et al. [61]. We cannot assume that mushrooms follow the exact mechanisms of plants but this is fundamental knowledge when there is no other report on fungi.

Phenolic compounds are the main antioxidant components in mushrooms. The composition of the phenolic profile of unfortified mushrooms was varied, with a very diverse profile of 10 acids (gallic, 2,5-DHBA, 4-HBA, vanillic, caffeic, syringic, *p*-coumaric, chlorogenic, ferulic, and sinapic acids for *P. ostreatus* to a very poor composition consisting of only gallic and trans-cinnamic acids for *P. nameko*). The obtained results show that there was great differentiation of content and composition of phenolic acids due to Fe-supplementation. Higher concentration of Fe generally resulted in the reduction in phenolic acid content, probably because different forms of iron have an inhibitory effect on the pathway of phenolic S formation. Additionally, different rates of decrease in phenolic acid content may be related to the forms of Fe added to substrates and their effect on the metabolic pathway. The content of some phenolic acids, including *p*-coumaric acid, increased for Fe-supplemented mushrooms. *P*-coumaric acid is well-known for its antioxidant activity and ability to scavenge free radicals. The content increase may be related to the Fe addition to the substrate and the induction of free radicals [62,63,64]. Knowledge of the effect of Fe-fortification on phenolic acids profile in mushrooms is limited. Vieira et al. [24] reported that Fe-enrichment did not affect phenolic content. In our previous study [50], we reported that Fe-supplementation of *P. nameko* brought about a qualitative and quantitative response in phenolic acid composition.

However, other experiments carried out on vegetables, fruits, and grains and Fe-enrichment have produced highly different results. It has been documented that Fe-fortification of various cultivars of lettuce did not result in an increase in the content of total phenolic content in every case [62]. A significant effect on total phenolic was achieved for Fe-biofortified (ferrous sulfate) germinated brown rice. However, the profile was not analyzed [65]. The authors showed that the increase of phenolic was up to nearly four-times in comparison to brown rice seeds. Experiments on Fe-biofortified carioca bean revealed a reduction in some polyphenols (epicatechin and quercetin 3-glucoside) [66], while Fe-biofortified black bean showed an elevation of some (gallic acid, kaempferol 3-glucoside, quercetin 3-glucoside, catechin, and myricetin) and a reduction in other (caffeic acid) phenolic compounds [67]. Considering the ability to improve phenolic content in mushrooms, it is worth considering the bioavailability of Fe in these products because it is believed that some phenolic compounds may be inhibitors of iron bioavailability [68,69].

This study confirmed that the fruiting bodies of mushrooms, depending on the species and type, can create diverse content and profile of the analyzed organic acids [70]. This is even more critical information, bearing in mind that in the case of organic acids, the profile and composition in mushroom fruiting bodies are key factors influencing their organoleptic characteristics and are early measures of reducing cellular oxidative load [71], and their chemical structure, profile, and content in fruiting bodies may determine their antioxidant capacity [72]. It should not be forgotten that organic acids have chelating abilities that significantly impact the Fe content in fruiting bodies. A number of studies similar to ours have reported that mushroom fruiting bodies, including *Pleurotus* and *Agaricus* [40,41,73], are able to effectively accumulate metals such as Fe, Pb, and Zn and carry them further to the fruiting bodies to store them in cell vacuoles as complexes with organic acids such as citrate, acetate, oxalate, and fumarate [41]. Several studies have also shown that organic acids present in mushroom extracts such as oxalic, acetic, malic, and citric have a high affinity for metal ions [74]. The creation of acetic acid in *Pleurotus* fruiting bodies was influenced by supplemented Fe salts. The Fe sources that allowed the highest amounts of acetic acid were FeSO_4_ and FeHBED salts for *P. ostreatus* and *P. eryngi*, although in the case of this species, the content of acetic acid was lower. In all the *P. nameko* samples, it appeared only in vestigial amounts, except for FeCl_3_ in which acetic acid was a dominant acid. Thus, in a general way, the accumulation of acetic acid appears to relate to the assimilation of Fe. Moreover, the present study confirmed the results obtained for *P. nameko* described by Budzyńska et al. [50], where supplementation with Fe was the cause of a lower synthesis of organic acids compared to the control.

The addition of Fe salts seems to exert inhibitory effects on oxalic production. This is important as calcium oxalate is the principal component of kidney stones and can be directly absorbed by the gut despite its insolubility. Although oxalic acid concentrations were low in the analyzed fruiting bodies, their presence can be further reduced or even prevented by applying different supplemented salts that can benefit humans [75].

## 4. Materials and Methods

### 4.1. Microorganisms and Spawn

In the experiment, three strains of mushrooms were used: B127 (*P. eryngii*), PN04 (*P. nameko*), and Spoppo—(*P. ostreatus*). B127 and PN04 strains were obtained from the Mushroom Collection of Poznań University of Life Science (Department of Vegetable Crops) and *P. ostreatus* from the Sylvan Company. The spawn for substrate inoculation was prepared with the method described by Stamets [76].

### 4.2. Substrate Preparation

Substrate from a mixture of alder and beech sawdust (1:1 vol.) was used for *P. eryngii* and *P. nameko* cultivation. The substrate was additionally supplemented with wheat bran to the amount of 20%, cornmeal 5%, soy meal 5%, gypsum 1% and saccharose 1%. It was mixed with an LPM 20 stirrer (Glass, Paderborn, Germany) and moistened with Fe compounds dissolved in distilled water. Ten experimental systems were prepared in this way with Fe added to the substrate as (1) iron (III) chloride hexahydrate (FeCl_3_ 6H_2_O); (2) iron (II) sulfate heptahydrate (FeSO_4_ 7H_2_O); and (3) bis(2-hydroxybenzyl)ethylenediamine diacetic acid, ferric potassium complex (FeHBED) (Merck KGaA, Darmstadt, Germany) separately in 3 concentrations: (a) 5, (b) 10, and (c) 50 mM including a control system free of Fe addition. After mixing with the solutions, the substrates had a humidity of 60%. The substrates were then placed in polypropylene bottles of 0.85 dm^3^ volume. Each bottle was filled with 450 g of the substrate and closed and covered with PP filters (class F-9, Filtropol, Poland). The substrates were sterilized at a temperature of 121 °C for one hour, after which they were cooled to a temperature of 25 °C. The substrates were then inoculated with 10 g of spawn (on wheat grain) per bottle.

The substrate for *P. ostreatus* was prepared from wheat straw to cut into chaff 2–4 cm long. The chaff was mixed with a solution of iron compounds dissolved in distilled water. The type and concentrations of the formulations used were the same as in the case of *P. eryngii* and *P. nameko*. The substrates were packed in 5 L polypropylene sacs, sterilized at a temperature of 121 °C for one hour, and then cooled down to a temperature of 25 °C. The substrates were then mixed with grain mycelium (on wheat grain), which constituted 3% of the wet weight of the substrate and placed in bags of perforated polyethylene foil. Each bag contained 1 kg of the substrate.

Incubation was conducted at a temperature of 25 °C and 80–85% air relative humidity until the substrate became completely covered with mycelium. Next, the bottles with removed covers and bags were placed in a cultivation room. For fructification, air relative humidity was maintained at 85–90% and temperature at 15 ± 1 °C. The cultivation was additionally lit with fluorescent light of 500 lx intensity 12 h per day. The growth room was aerated in such a way as to maintain CO_2_ concentration below 1000 ppm. Carpophores were harvested successively as they matured. For *P. eryngii* and *P. ostreatus*, the first and the second crop were collected. The crop included whole fruiting bodies.

### 4.3. Determination of Fe, Ca, K, Mg, Na, Mn and S

#### 4.3.1. Sample Preparation for Element Analysis

Samples were dried at 45 ± 5 °C for 96 h in an electric oven (SLW 53 STD, Pol-Eko, Poland) and ground in a laboratory Cutting Boll Mill PM 200 (Retsch GmbH, Haan, Germany). The microwave sample preparation system Mars 6 (CEM, Matthews, NC, USA) was used for sample digestion. Accurately weighed 0.500 ± 0.001 g of a dry sample was digested by 5 mL of concentrated nitric acid (Suprapure, Merck, Germany) in closed Teflon containers in the microwave sample preparation system (180 °C, 20 min ramp time, 20 min heating time, and 20 min cooling). After digestion, samples were filtered and diluted with water to a final volume of 10.0 mL. Each sample was analyzed in triplicate using the whole sample preparation procedure.

#### 4.3.2. Instruments and Chemicals

The inductively coupled plasma optical emission spectrometer Agilent 5110 ICP-OES (Agilent, Santa Clara, CA, USA) was used for Fe, Ca, K, Mg, Na, Mn, and S determination. The plasma’s synchronous vertical dual view (SVDV) was accomplished with dichroic spectral combiner (DSC) technology, which allowed the axial and radial view to be analyzed simultaneously. The common instrumental conditions were used: radio frequency (RF) power 1.2 kW, nebulizer gas flow 0.7 L min^−1^, auxiliary gas flow 1.0 L min^−1^, plasma gas flow 12.0 L min^−1^, Charge Coupled Device (CCD) temperature −40 °C, viewing height for radial plasma observation 8 mm, accusation time 5 s, and 3 replicates. The wavelengths were: 238.204 nm for Fe (axial view), 422.673 nm for Ca (radial view), 285.213 nm for Mg (axial view), 766.491 nm for K (radial view), 589.592 nm for Na (radial view), 257.610 nm for Mn (axial view), and 181.972 nm for S (axial view), respectively.

#### 4.3.3. Quality Assurance and Quality Control (QA/QC) of Samples

The detection limits were determined as 3 sigma criteria and were on the level of 0.0X mg kg^−1^ DW for all elements determined (0.02 mg kg^−1^ for Fe, 0.03 mg kg^−1^ for Ca, 0.03 mg kg^−1^ for K, 0.01 mg kg^−1^ for Mg, 0.03 mg kg^−1^ for Na, 0.01 mg kg^−1^ for Mn, and 0.4 mg kg^−1^ for S, respectively). The traceability was checked by analysis of the reference materials CRM S-1—loess soil; CRM NCSDC (73349)—bush branches and leaves; CRM 2709—soil; and the recovery (80–120%) was acceptable for most of the elements determined. For uncertified elements, the recovery was defined using the standard addition method.

### 4.4. Sample Preparation and Analysis of Phenolic Compounds

The extraction of phenolic acids from mushroom samples and UPLC determination was according to Gąsecka et al. [40]. The samples were mixed with 80% methanol and were shaken for 8 h at room temperature. They were then centrifuged and evaporated to dryness at 40 °C. For further analysis, the extracts were redissolved in 1 mL of 80% methanol and filtered. An ACQUITY UPLC H-Class System and PDA eλ Detector (Waters Corporation, Milford, MA, USA) were used for phenolic acid identification. The separation of the acids was conducted on an Acquity UPLC BEH C18 column (2.1 mm×150 mm, 1.7 µm, Waters) thermostated at 35 °C. Gradient elution was performed with water and acetonitrile (both containing 0.1% formic acid, pH = 2) at a 0.4 mL/min flow rate.

### 4.5. Sample Preparation and Organic Acid Analysis

The extraction of organic acids from mushroom samples was according to Magdziak et al. [41], *Pleurotus* species samples were extracted by water in an ultrasound bath (320 W, 2 × 15 min, at ambient temperature) (Bandelin Sonorex RK 100, Berlin, Germany), centrifuged at 3000 rpm with a Universal 320 R centrifuge (Hettich, Tuttlingen, Germany) and evaporated to dryness. The samples were stored at −20 °C before analyses. For UPLC analyses, the extracts were redissolved in 1 mL of deionized water (Mili-Q, Millipore, Molsheim, France) and filtered. The UPLC analysis of organic acids was performed identically as described for phenolic compounds.

### 4.6. Statistical Analysis

Standard statistical analysis including analysis of variance (one-way ANOVA) and post-hoc Tukey’s HSD test, were used to evaluate the significant changes between the analyzed parameters in combinations. Two-way ANOVA was used to evaluate the significance of “salt” and “concentration” single and fixed effects on phenolic and organic acid content.

## 5. Conclusions

After pioneering research with three basidiomycete mushroom species grown in culture media with three different Fe salts and three concentrations of each metal form, we concluded that Fe bioaccumulation efficiency is significantly influenced by metal concentration in the substrate, mushroom species, and the chemical form of Fe as an additive. Therefore, it is impossible to arrive at one conclusion about the ideal species for supplementation, the best concentration, or the ideal form of Fe. Each case should be treated individually. When choosing the species we would like to enrich, we must find the best conditions (form and concentration in the medium) to obtain the highest efficiency. Knowing the possibilities, we are on a straight path to applying such biofortification in practice. Based solely on our results, we recommend that the most effective concentration of the additive during Fe fortification is 50 mM. Furthermore, the proper selection of Fe salt and its concentration gives the opportunity not only to produce fruit bodies rich in Fe but also with a higher content of phenolic acids than un-biofortified mushrooms.

## Figures and Tables

**Table 1:** Content (mg kg^−1^ DW) of selected elements in *Pleurotus eryngii* fruit bodies after the I and II crop.

Experimental System	Ca	K	Mg	Mn	Na	S
	I Crop					
Control	361 ^e^ ± 37.4	10,076 ^e^ ± 1160	583 ^cd^ ± 49.2	2.61 ^c^ ± 0.21	153 ^e^ ± 15.1	4099 ^a^ ± 476
1a	562 ^cd^ ± 57.8	12,460 ^cde^ ± 1351	637 ^bcd^ ± 60.7	5.51 ^ab^ ± 0.40	152 ^e^ ± 14.9	4355 ^a^ ± 318
1b	791^b^ ± 70.5	16,168 ^abc^ ± 1543	784 ^ab^ ± 89.2	5.35 ^ab^ ± 0.37	181 ^de^ ± 13.8	4451 ^a^ ± 499
1c	860^b^ ± 72.1	18,805 ^a^ ± 1776	908 ^a^ ± 64.5	6.38 ^a^ ± 0.37	180 ^de^ ± 16.2	4814 ^a^ ± 514
2a	566 ^cd^ ± 49.4	11,552 ^de^ ± 983	562 ^d^ ± 49.3	4.38 ^b^ ± 0.30	180 ^de^ ± 7.50	3949 ^a^ ± 262
2b	1131 ^a^ ± 121	16,835 ^ab^ ± 1052	752 ^abc^ ± 70.8	5.98 ^a^ ± 0.55	238 ^bc^ ± 17.2	4676 ^a^ ± 346
2c	680 ^bc^ ± 74.2	14,525 ^bcd^ ± 1561	744 ^abc^ ± 47.0	5.64 ^a^ ± 0.56	285 ^ab^ ± 19.4	4699 ^a^ ± 301
3a	818 ^b^ ± 82.8	14,862 ^abcd^ ± 1367	729 ^bcd^ ± 45.3	6.09 ^a^ ± 0.36	212 ^cd^ ± 19.5	4368 ^a^ ± 374
3b	704 ^bc^ ± 66.6	14,827 ^abcd^ ± 1543	764 ^ab^ ± 55.4	5.23 ^ab^ ± 0.37	270 ^ab^ ± 29.8	4256 ^a^ ± 248
3c	695 ^bc^ ± 48.4	14,886 ^abcd^ ± 1563	761 ^ab^ ± 46.3	5.65 ^a^ ± 0.59	292 ^a^ ± 16.6	4547 ^a^ ± 306
	II crop					
Control	242 ^f^ ± 26.2	9005 ^d^ ± 413	365 ^d^ ± 27.8	2.47 ^c^ ± 0.19	138 ^e^ ± 9.89	836 ^d^ ± 87.9
1a	743 ^d^ ± 75.3	10,511 ^d^ ± 1181	379 ^d^ ± 32.7	3.90 ^b^ ± 0.44	266 ^c^ ± 19.6	806 ^d^ ± 47.0
1b	658 ^de^ ± 67.5	21,406 ^b^ ± 1394	677 ^b^ ± 45.5	4.33 ^b^ ± 0.43	263 ^cd^ ± 19.3	1335 ^cd^ ± 86.5
1c	1335 ^b^ ± 121	28,030 ^a^ ± 2860	930 ^a^ ± 88.3	5.67 ^a^ ± 0.33	530 ^a^ ± 59.9	1634 ^cd^ ± 135
2a	446 ^ef^ ± 28.1	10,602 ^d^ ± 1092	500 ^cd^ ± 65.9	2.55 ^c^ ± 0.16	191^de^ ± 16.2	1749 ^c^ ± 126
2b	480 ^ef^ ± 24.4	10,463 ^d^ ± 718	610 ^bc^ ± 44.9	2.44 ^c^ ± 0.25	166 ^e^ ± 9.89	3912 ^ab^ ± 458
2c	1566 ^a^ ± 82.6	15,513 ^c^ ± 1322	689 ^b^ ± 48.4	2.95 ^c^ ± 0.25	377 ^b^ ± 21.3	4608 ^a^ ± 444
3a	263 ^f^ ± 16.8	9121 ^d^ ± 526	504 ^cd^ ± 55.8	2.56 ^c^ ± 0.11	193^de^ ± 22.0	3579 ^b^ ± 356
3b	515 ^de^ ± 36.3	10,962 ^d^ ± 932	579 ^bc^ ± 58.8	2.66 ^c^ ± 0.17	149 ^e^ ± 8.38	3872 ^ab^ ± 333
3c	1008 ^c^ ± 84.3	10,159 ^d^ ± 1059	556 ^bc^ ± 59.4	2.98 ^c^ ± 0.27	156 ^e^ ± 8.36	4337 ^ab^ ± 282

Mean values (*n* = 3) ± standard deviations; identical superscripts (^a^, ^b^, ^c^…) denote no significant (*p* < 0.05) difference between mean values in column according to Tukey’s HDS test (ANOVA).

**Table 2:** Content (mg kg^−1^ DW) of selected elements in *Pleurotus ostreatus* fruit bodies after the I and II crop.

Experimental System	Ca	K	Mg	Mn	Na	S
	I Crop					
Control	538 ^d^ ± 42.7	16,265 ^de^ ± 1393	637 ^de^ ± 36.4	1.97 ^e^ ± 0.20	90.2 ^ef^ ± 7.68	576 ^e^ ± 64.4
1a	611 ^cd^ ± 43.7	19,985 ^cd^ ± 2104	839 ^cd^ ± 80.8	2.29 ^cde^ ± 0.18	117 ^de^ ± 12.7	421 ^e^ ± 12.5
1b	535 ^d^ ± 34.1	17,887 ^cd^ ± 1010	860 ^c^ ± 97.2	2.36 ^cde^ ± 0.14	96.0 ^def^ ± 4.42	444 ^e^ ± 21.8
1c	623 ^cd^ ± 46.1	19,765 ^cd^ ± 2124	946 ^bc^ ± 70.5	2.45 ^cde^ ± 0.18	160 ^bc^ ± 16.8	365 ^e^ ± 35.4
2a	758 ^c^ ± 78.3	15,467 ^de^ ± 1018	737 ^cde^ ± 47.1	2.35 ^cde^ ± 0.21	62.5 ^f^ ± 7.26	1480 ^d^ ± 14.4
2b	973 ^ab^ ± 54.6	16,417 ^de^ ± 1701	800 ^cd^ ± 52.2	2.14 ^de^ ± 0.16	160 ^bc^ ± 18.2	4183 ^a^ ± 475
2c	1115 ^a^ ± 100	31,057 ^a^ ± 2077	556^e^ ± 45.0	2.29 ^cde^ ± 0.23	194 ^ab^ ± 21.0	3896 ^ab^ ± 407
3a	778 ^bc^ ± 40.7	11,529 ^e^ ± 1221	1127 ^ab^ ± 83.8	2.81 ^bc^ ± 0.27	136 ^cd^ ± 11.2	4515 ^a^ ± 428
3b	967 ^ab^ ± 87.7	21,747 ^bc^ ± 1420	1162 ^ab^ ± 123	3.19 ^ab^ ± 0.20	190 ^ab^ ± 17.9	2053 ^c^ ± 136
3c	1011 ^a^ ± 107	26,084 ^b^ ± 2348	1268 ^a^ ± 84.0	3.74 ^a^ ± 0.18	205 ^a^ ± 16.3	3225 ^b^ ± 211
	II crop					
Control	583 ^c^ ± 41.3	14,685 ^c^ ± 1423	557 ^b^ ± 63.7	1.85 ^d^ ± 0.28	158 ^e^ ± 10.9	402 ^e^ ± 26.6
1a	707 ^b^ ± 60.9	15,000 ^c^ ± 1330	767 ^ab^ ± 57.5	2.62 ^abcd^ ± 0.19	161 ^e^ ± 15.8	392 ^e^ ± 37.0
1b	553 ^b^ ± 50.0	16,307 ^bc^ ± 1821	702 ^ab^ ± 60.8	2.17 ^cd^ ± 0.24	272 ^c^ ± 26.1	416 ^e^ ± 32.7
1c	590 ^b^ ± 56.5	16,564 ^bc^ ± 1896	745 ^ab^ ± 71.4	2.38 ^bcd^ ± 0.20	364 ^a^ ± 27.4	536 ^de^ ± 33.3
2a	953 ^a^ ± 97.3	21,206 ^ab^ ± 1520	821 ^a^ ± 79.7	2.36 ^bcd^ ± 0.25	193 ^de^ ± 11.1	659 ^cd^ ± 56.7
2b	942 ^a^ ± 79.9	21,217 ^ab^ ± 1402	825 ^a^ ± 80.4	2.06 ^d^ ± 0.21	344 ^ab^ ± 29.8	685 ^cd^ ± 49.5
2c	733 ^b^ ± 70.6	23,363 ^a^ ± 2673	838 ^a^ ± 83.5	2.29 ^bcd^ ± 0.24	304 ^abc^ ± 24.2	587 ^d^ ± 46.3
3a	559 ^b^ ± 42.2	19,369 ^abc^ ± 1346	779 ^ab^ ± 86.4	2.79 ^ab^ ± 0.15	291 ^bc^ ± 30.2	917 ^ab^ ± 68.5
3b	690 ^b^ ± 22.3	20,128 ^ab^ ± 1672	727 ^ab^ ± 79.4	3.02 ^a^ ± 0.18	238 ^cd^ ± 22.6	784 ^bc^ ± 66.3
3c	1031 ^a^ ± 70.7	21,731 ^a^ ± 1833	593 ^b^ ± 56.3	2.73 ^abc^ ± 0.22	343 ^ab^ ± 25.6	1017 ^a^ ± 97.9

Mean values (*n* = 3) ± standard deviations; identical superscripts (^a^, ^b^, ^c^….) denote no significant (*p* < 0.05) difference between mean values in column according to Tukey’s HDS test (ANOVA).

**Table 3:** Content (mg kg^−1^ DW) of selected elements in *Pholiota nameko* fruit bodies.

Experimental System	Ca	K	Mg	Mn	Na	S
Control	208 ^ef^ ± 15.4	10,675 ^a^ ± 1002	626 ^de^ ± 60.4	6.45 ^cd^ ± 0.55	215 ^e^ ± 20.5	6076 ^abc^ ± 333
1a	367 ^ab^ ± 28.4	10,293 ^a^ ± 653	677 ^cde^ ± 73.6	8.89 ^ab^ ± 0.78	457 ^a^ ± 31.4	5268 ^c^ ± 505
1b	331 ^bc^ ± 30.1	11,828 ^a^ ± 585	1074 ^ab^ ± 86.0	8.24 ^abc^ ± 0.71	372 ^bc^ ± 24.6	6493 ^abc^ ± 544
1c	356 ^b^ ± 30.2	11,430 ^a^ ± 974	909 ^b^ ± 88.2	9.14 ^a^ ± 0.88	381 ^b^ ± 28.1	6011 ^abc^ ± 576
2a	221 ^ef^ ± 13.0	11,977 ^a^ ± 900	849 ^bcd^ ± 82.4	7.14 ^bcd^ ± 0.52	265 ^de^ ± 18.1	6189 ^abc^ ± 633
2b	202 ^f^ ± 16.8	12,549 ^a^ ± 824	1063 ^ab^ ± 75.8	7.46 ^abcd^ ± 0.72	277 ^de^ ± 24.7	7192 ^a^ ± 688
2c	432 ^a^ ± 37.0	11,488 ^a^ ± 1244	1237 ^a^ ± 108	6.38 ^cd^ ± 0.58	253 ^de^ ± 23.8	6300 ^abc^ ± 375
3a	257 ^def^ ± 18.9	11,586 ^a^ ± 991	559 ^e^ ± 40.9	6.40 ^cd^ ± 0.57	373 ^bc^ ± 21.1	5949 ^abc^ ± 462
3b	273 ^cde^ ± 23.0	10,932 ^a^ ± 930	863 ^bc^ ± 75.3	6.18 ^d^ ± 0.67	364 ^bc^ ± 23.0	6967 ^ab^ ± 396
3c	314 ^bcd^ ± 26.8	10,012 ^a^ ± 592	871 ^bc^ ± 68.4	6.15 ^d^ ± 0.54	305 ^cd^ ± 25.5	5655 ^bc^ ± 429

Mean values (*n* = 3) ± standard deviations; identical superscripts (^a^, ^b^, ^c^….) denote no significant (*p* < 0.05) difference between mean values in column according to Tukey’s HDS test (ANOVA).

**Table 4:** Phenolic acids profile (µg g^−1^ DW) of fruiting bodies of the mushroom species under different Fe salts supplementation and concentrations (mean value ± standard deviation).

Experimental System	Control	1a	1b	1c	2a	2b	2c	3a	3b	3c	*p*-Value (S × C)
*P. ostreatus*											
gallic	42.0 ^b^ ± 3.03	82.0 ^a^ ± 3.04	2.22 ^g^ ± 0.08	2.05 ^g^ ± 0.15	3.21 ^fg^ ± 0.32	31.8 ^c^ ± 0.79	22.1 ^d^ ± 1.18	7.41 ^ef^ ± 0.34	9.33 ^e^ ± 0.31	23.2 ^d^ ± 2.23	0.000
2,5-dihydroxybenzoic	12.4 ^a^ ± 1.34	bDL	bDL	bDL	bDL	9.19 ^b^ ± 0.54	2.23 ^c^ ± 0.13	0.910 ^d^ ± 0.08	10.1 ^b^ ± 0.39	bDL	0.000
4-hydroxybenzoic	75.3 ^b^ ± 3.57	108 ^a^ ± 6.36	6.68 ^ef^ ± 0.42	2.71 ^f^ ± 0.5	7.26 ^ef^ ± 0.28	59.9 ^c^ ± 1.29	22.5 ^d^ ± 1.91	5.39 ^ef^ ± 0.45	11.8 ^e^ ± 1.76	22.0 ^d^ ± 2.26	0.000
vanillic	21.0 ^b^ ± 2.24	43.3 ^a^ ± 2.37	5.55 ^de^ ± 0.28	4.43 ^de^ ± 0.26	5.49 ^de^ ± 0.24	7.87 ^cd^ ± 0.30	11.1 ^c^ ± 2.01	3.25 ^e^ ± 0.17	11.4 ^c^ ± 2.48	11.5 ^c^ ± 0.70	0.000
caffeic	0.700 ^b^ ± 0.03	bDL	0.280 ^c^ ± 0.02	bDL	bDL	1.59 ^a^ ± 0.12	bDL	0.600 ^b^ ± 0.02	bDL	0.220 ^c^ ± 0.03	0.000
syringic	12.6^b^ ± 1.89	44.8 ^a^ ± 2.18	0.270 ^e^ ± 0.23	0.31 ^e^ ± 0.02	0.57 ^de^ ± 0.05	6.37 ^c^ ± 0.34	3.24 ^d^ ± 0.30	1.33 ^de^ ± 0.17	1.11 ^de^ ± 0.18	5.98 ^c^ ± 0.018	0.000
*p*-coumaric	bDL	2.61 ^a^ ± 0.17	0.850 ^b^ ± 0.07	0.14 ^e^ ± 0.02	bDL	0.31 ^cd^ ± 0.02	0.40 ^c^ ± 0.02	0.21 ^d^ ± 0.02	0.160 ^de^ ± 0.02	0.680 ^b^ ± 0.05	0.000
chlorogenic	1.37 ^b^ ± 0.15	8.66 ^a^ ± 0.42	bDL	bDL	0.20 ^b^ ± 0.02	1.55 ^b^ ± 0.12	0.60 ^b^ ± 0.03	1.21 ^b^ ± 1.60	0.230 ^b^ ± 0.04	1.93 ^b^ ± 2.56	0.000
ferulic	0.470 ^c^ ± 0.06	2.37 ^a^ ± 0.26	bDL	bDL	bDL	0.20 ^d^ ± 0.01	bDL	0.590 ^c^ ± 0.02	0.470 ^c^ ± 0.02	0.92 ^b^ ± 0.03	0.000
sinapic	27.8 ^a^ ± 2.13	3.79 ^b^ ± 0.27	0.250 ^c^ ± 0.01	0.22 ^c^ ± 0.02	0.50 ^c^ ± 0.64	0.46 ^c^ ± 0.03	0.25 ^c^ ± 0.03	0.12 ^c^ ± 0.03	0.200 ^c^ ±0.08	0.160 ^c^ ± 0.01	0.000
sum of acids	194^b^ ± 13.51	295 ^a^ ± 9.69	16.1 ^f^ ± 0.69	9.75 ^f^ ± 0.60	17.2 ^f^ ± 0.90	119 ^c^ ± 1.61	62.6 ^d^ ± 2.07	21.0 ^f^ ± 1.61	44.8 ^e^ ± 1.34	66.7 ^d^ ± 7.31	0.000
*P. eryngii*											
gallic	6.99^c^ ± 0.20	14.3 ^a^ ± 1.10	1.57 ^e^ ± 0.18	bDL	8.33 ^b^ ± 0.38	3.12 ^d^ ± 0.13	bDL	6.48 ^c^ ± 0.33	3.39 ^d^ ± 0.42	0.780 ^ef^ ± 0.03	0.000
vanillic	10.0^c^ ± 0.90	8.17 ^d^ ± 0.13	0.590 ^f^ ± 0.02	bDL	23.8 ^a^ ± 0.73	11.5 ^c^ ± 0.92	18.4 ^b^ ± 1.09	4.57 ^e^ ± 0.38	4.56 ^e^ ± 0.18	0.580 ^f^ ± 0.03	0.000
caffeic	0.16 ^d^ ± 0.05	0.290 ^d^ ± 0.08	bDL	bDL	bDL	1.07 ^b^ ± 0.04	1.47 ^a^ ± 0.08	0.390 ^c^ ± 0.02	bDL	bDL	0.000
syringic	2.14 ^b^ ± 0.77	2.07 ^b^ ± 0.10	0.880 ^d^ ± 0.04	bDL	2.80 ^a^ ± 0.16	0.800 ^d^ ± 0.07	0.940 ^d^ ± 0.06	1.24 ^c^ ± 0.13	bDL	bDL	0.000
*p*-coumaric	0.450 ^c^ ± 0.05	0.260 ^e^ ± 0.02	0.810 ^a^ ± 0.02	bDL	0.570 ^b^ ± 0.03	0.180 ^e^ ± 0.02	0.340 ^d^ ± 0.02	bDL	bDL	bDL	0.000
chlorogenic	0.780 ^a^ ± 0.05	0.630 ^bc^ ± 0.04	0.540 ^d^ ± 0.02	bDL	0.680 ^ab^ ± 0.02	0.720 ^ab^ ± 0.07	0.660 ^b^ ± 0.04	0.160 ^e^ ± 0.02	bDL	bDL	0.000
ferulic	0.770 ^a^ ± 0.03	0.650 ^b^ ± 0.02	0.140 ^d^ ± 0.02	bDL	0.450 ^c^ ± 0.04	0.120 ^d^ ± 0.04	0.150 ^d^ ± 0.02	0.120 ^d^ ± 0.01	bDL	bDL	0.000
sinapic	0.570 ^a^ ± 0.06	0.190 ^c^ ± 0.02	bDL	bDL	0.240 ^c^ ± 0.00	0.310 ^b^ ± 0.02	bDL	0.210 ^c^ ± 0.01	bDL	bDL	0.000
sum of acids	21.9 ^c^ ± 0.63	26.6 ^b^ ± 1.01	4.53 ^g^ ± 0.12	bDL	36.9 ^a^ ± 0.97	17.9 ^d^ ± 0.93	22.0 ^c^ ± 1.17	13.2 ^e^ ± 0.67	7.95 ^f^ ± 0.46	1.36 ^d^ ± 0.05	0.000
*P. nameko*											
gallic	12.8 ^a^ ± 0.65	4.74 ^b^ ± 0.26	1.91 ^d^ ± 0.23	2.63 ^c^ ± 0.21	bDL	bDL	bDL	bDL	bDL	bDL	0.000
4-hydroxybenzoic	bDL	2.79 ^c^ ± 0.61	9.09 ^a^ ± 0.20	3.73 ^b^ ± 0.23	bDL	bDL	bDL	bDL	bDL	bDL	0.000
t-cinnamic	6.69 ^a^ ± 0.61	1.51 ^c^ ± 0.06	0.220 ^f^ ± 0.05	0.890 ^de^ ± 0.02	3.97 ^b^ ± 0.16	2.44 ^ef^ ± 0.02	0.610 ^ef^ ± 0.02	1.82 ^c^ ± 0.18	0.590 ^ef^ ± 0.03	1.33 ^cd^ ± 0.33	0.000
sum	19.5 ^a^ ± 1.06	9.03 ^bc^ ± 3.85	11.2 ^b^ ± 0.47	7.26 ^cd^ ± 0.16	3.97 ^de^ ± 0.16	0.240 ^f^ ± 0.02	0.610 ^ef^ ± 0.02	1.83 ^ef^ ± 0.18	0.580 ^ef^ ± 0.03	1.33 ^ef^ ± 0.33	0.004

bDL—below detection limit; identical superscripts (^a^, ^b^, ^c^….) in row denote no significant differences between means according to a post-hoc Tukey’s HSD test at α = 95% following two-way ANOVA (*n* = 3).

**Table 5:** Organic acids profile and content (µg g^−1^ DW) of fruiting bodies of mushroom species under different Fe salts supplementation and concentrations.

Experimental System	Control	1a	1b	1c	2a	2b	2c	3a	3b	3c
*P. ostreatus*										
oxalic	bDL	3.74 ^a^ ± 0.13	0.140 ^f^ ± 0.01	0.170 ^ef^ ± 0.01	0.460 ^d^ ± 0.02	2.32 ^b^ ± 0.08	bDL	1.29 ^c^ ± 0.05	0.350 ^de^ ± 0.01	bDL
quinic	16.7 ^a^ ± 2.59	bDL	bDL	bDL	bDL	bDL	bDL	bDL	bDL	bDL
malic	0.630 ^de^ ± 0.02	1.44 ^d^ ± 0.05	bDL	bDL	bDL	0.870 ^de^ ± 0.03	19.6 ^a^ ± 0.69		8.11 ^c^ ± 0.28	17.6 ^b^ ± 1.61
malonic	1.89^c^ ± 0.07	3.84 ^a^ ± 0.14	0.400 ^f^ ± 0.01	0.160 ^g^ ± 0.01	0.270 ^fg^ ± 0.01	3.11 ^b^ ± 0.11	1.26 ^d^ ± 0.04	1.90 ^c^ ± 0.07	0.860 ^e^ ± 0.03	0.110 ^g^ ± 0.01
lactic	bDL	bDL	bDL	bDL	bDL	bDL	0.010 ^a^ ± 0.01	bDL	bDL	bDL
citic	bDL	bDL	0.630 ^b^ ± 0.02	0.700 ^a^ ± 0.02	bDL	bDL	bDL	bDL	bDL	bDL
acetic	45.5 ^a^ ± 1.60	40.8 ^b^ ± 1.43	4.97 ^ef^ ± 0.17	2.74 ^f^ ± 0.10	9.07 ^e^ ± 0.32	43.4 ^ab^ ± 1.53	46.2 ^a^ ± 1.63	21.0 ^d^ ± 0.74	31.0 ^c^ ± 1.09	47.3 ^a^ ± 1.67
fumaric	0.250 ^b^ ± 0.01	0.230 ^c^ ± 0.01	bDL	4.38×10^−4 e^ ± 1×10^−6^	bDL	0.200 ^d^ ± 0.01	0.320 ^a^ ± 0.04	bDL	bDL	bDL
succinic	bDL	0.790 ^c^ ± 0.03	bDL	bDL	0.170 ^d^ ± 0.01	bDL	bDL	bDL	6.45 ^b^ ± 0.23	8.63 ^a^ ± 0.30
sum	64.9 ^b^ ± 2.28	50.8 ^c^ ± 1.79	6.15 ^ef^ ± 0.22	3.78 ^f^ ± 0.13	9.96 ^e^ ± 0.35	49.9 ^c^ ± 1.75	67.4 ^b^ ± 2.37	24.2 ^d^ ± 1.85	46.7 ^c^ ± 1.64	73.8 ^a^ ± 2.59
*P.eryngii*										
oxalic	0.160 ^gh^ ± 0.01	1.28 ^c^ ± 0.04	0.230 ^g^ ± 0.01	bDL	2.80 ^a^ ± 0.09	1.93 ^b^ ± 0.07	0.650 ^e^ ± 0.02	0.930 ^d^ ± 0.03	0.500 ^f^ ± 0.01	0.070 ^hi^ ± 0.01
quinic	bDL	bDL	bDL	bDL	bDL	bDL	bDL	bDL	bDL	bDL
malic	10.7 ^a^ ± 1.37	0.520 ^e^ ± 0.02	bDL	bDL	bDL	5.32 ^b^ ± 0.48	2.28 ^d^ ± 0.08	4.36 ^c^ ± 0.35	0.580 ^e^ ± 0.02	0.900 ^e^ ± 0.03
malonic	0.730 ^e^ ± 0.02	2.28 ^a^ ± 0.09	0.130 ^h^ ± 0.01	bDL	0.120 ^h^ ± 0.01	2.13 ^b^ ± 0.07	1.21 ^c^ ± 0.04	0.500 ^f^ ± 0.01	0.900 ^d^ ± 0.03	0.330 ^g^ ± 0.01
lactic	bDL	bDL	bDL	bDL	bDL	bDL	bDL	bDL	bDL	bDL
citric	bDL	bDL	3.18 ^a^ ± 0.11	bDL	bDL	bDL	bDL	bDL	2.69 ^b^ ± 0.09	bDL
acetic	23.7 ^c^ ± 1.83	29.3 ^b^ ± 1.03	bDL	bDL	32.8 ^a^ ± 1.15	25.9 ^c^ ± 0.91	15.6 ^d^ ± 0.55	33.6 ^a^ ± 1.18	1.59 ^e^ ± 0.06	1.72^e^ ± 0.06
fumaric	bDL	bDL	bDL	bDL	bDL	bDL	bDL	bDL	bDL	bDL
succinic	bDL	bDL	bDL	bDL	bDL	bDL	bDL	bDL	bDL	bDL
sum	35.3 ^b^ ± 1.24	33.4 ^b^ ± 1.17	3.55 ^d^ ± 0.12	bDL	35.7 ^b^ ± 1.26	35.2 ^b^ ± 1.24	19.8 ^c^ ± 0.70	39.4 ^a^ ± 1.39	6.20 ^d^ ± 0.22	3.03 ^de^ ± 0.11
*P. nameko*										
oxalic	1.16 ^a^ ± 0.04	0.050 ^b^ ± 0.01	0.070 ^b^ ± 0.01	0.070 ^b^ ± 0.01	bDL	bDL	bDL	bDL	bDL	bDL
quinic	bDL	bDL	bDL	bDL	bDL	bDL	bDL	bDL	bDL	bDL
malic	bDL	0.530 ^b^ ± 0.02	1.16 ^a^ ± 0.04	1.19 ^a^ ± 0.04	bDL	bDL	bDL	bDL	bDL	0.260 ^c^ ± 0.01
malonic	0.840 ^a^ ± 0.03	0.110 ^b^ ± 0.01	0.130 ^b^ ± 0.01	0.140 ^b^ ± 0.01	bDL	bDL	bDL	bDL	bDL	bDL
lactic	bDL	bDL	bDL	bDL	bDL	bDL	bDL	bDL	bDL	bDL
cirtic	2.42 ^a^ ± 0.08	0.170 ^b^ ± 0.01	bDL	bDL	bDL	bDL	bDL	bDL	bDL	bDL
acetic	11.7 ^a^ ± 0.42	0.350 ^c^ ± 0.01	2.29 ^b^ ± 0.08	2.19 ^b^ ± 0.07	bDL	bDL	bDL	bDL	bDL	0.160 ^c^ ± 0.01
fumaric	0.11 ^a^ ± 3.99×10^−3^	bDL	bDL	0.040 ^b^ ± 1.30×10^−3^	bDL	bDL	bDL	bDL	bDL	bDL
succinic	bDL	bDL	bDL	bDL	bDL	bDL	bDL	bDL	bDL	bDL
sum	16.3 ^a^ ± 1.57	1.22 ^c^ ± 1.04	3.64 ^b^ ± 0.12	3.63 ^b^ ± 0.12	bDL	bDL	bDL	bDL	bDL	0.420 ^d^ ± 0.01

bDL—below detection limit; identical superscripts (^a^, ^b^, ^c^….) in row denote no significant differences between means according to a post-hoc Tukey’s HSD test at α = 95% following a two-way ANOVA (*n* = 3).

## Data Availability

Not applicable.

## Sample Availability

Samples of the compounds are not available from the authors.
